# Unexpected Resection of Gray Ramus Communicans During Endoscopic Lumbar Surgery: A Report of Two Cases

**DOI:** 10.7759/cureus.102464

**Published:** 2026-01-28

**Authors:** Atsushi Shimizu, Kyohei Kin, Kento Takebayashi, Hisashi Koga

**Affiliations:** 1 Neurosurgery, Tokyo Woman's Medical University Hospital, Tokyo, JPN; 2 Neurosurgery, Okayama University Hospital, Okayama, JPN; 3 Neurosurgery, Iwai FESS Clinic, Tokyo, JPN

**Keywords:** aberrant nerve anatomy, anatomical variation, endoscopic lumbar discectomy, gray ramus communicans, intraoperative neural variation, lumbar disc herniation, minimally invasive spine surgery, peripheral nerve histopathology, sympathetic nerve variation, unexpected nerve structure

## Abstract

Encountering nerve-like structures that do not correspond to the typical course of the spinal nerve root during endoscopic lumbar discectomy is rare. We describe two cases in which fine nerve-like fibers were identified during posterolateral full-endoscopic lumbar discectomy using the outside-in technique and were subsequently confirmed histologically as neural tissue. Both patients underwent surgery for L3/4 foraminal disc herniation. During the removal of the fat tissue overlying the disc, thin, vine-like, whitish fibers were observed. No abnormalities were detected on motor evoked potentials (MEPs) or somatosensory evoked potentials (SEPs), and we did not identify any continuous central neural bundle.

In case 1, a preoperative fast imaging employing steady-state acquisition (FIESTA) MRI obtained 10 years earlier, before lumbar fusion, showed a fine branch arising from the L3 nerve root and coursing toward the disc, consistent with the intraoperative findings. Histopathology revealed ganglion-like structures in case 1 and predominantly unmyelinated nerve fibers in case 2. Histological examination revealed axonal structures clearly demonstrated by neurofilament staining, while Klüver-Barrera staining was negative, indicating the absence of myelinated fibers. These findings suggested that the excised fibers consisted predominantly of unmyelinated nerve fibers, consistent with a gray rami communicantes.

We review these anatomical findings and emphasize the need for awareness of such branches to prevent inadvertent nerve resection during endoscopic lumbar surgery.

## Introduction

Lumbar disc herniation is one of the most common spinal disorders, and full-endoscopic lumbar discectomy has become widely accepted as a minimally invasive surgical option. In transforaminal or posterolateral endoscopic discectomy, the surgeon accesses the disc through “Kambin’s triangle,” a safe working zone bounded superiorly by the exiting nerve root, inferiorly by the pedicle, and medially by the dura mater [1,2]. Because this region typically lacks neural structures other than conjoined nerve roots [2,3], encountering nerve-like structures within this zone is extremely uncommon and may present an unexpected intraoperative finding.

We report two cases in which nerve-like structures were visualized in locations inconsistent with known neural anatomy and subsequently confirmed histologically as nerve fibers. Both surgeries were performed using the posterolateral endoscopic lumbar discectomy approach employing the outside-in technique. This method, originally described by Hoogland in 2003 [4-6], expands the working space stepwise from the extraforaminal zone into the disc. Unlike the inside-out technique, which begins within the intradiscal space, the outside-in technique allows earlier direct visualization of structures surrounding the nerve root.

While this approach may facilitate neural protection by enabling early identification of the nerve root and foraminal structures, unrecognized anatomical variations, such as rami communicantes, may pose a risk of encountering unexpected neural fibers during needle placement or sheath insertion. Therefore, detailed preoperative understanding of neural anatomy and cautious intraoperative manipulation are essential [7].

This manuscript is based on a presentation delivered at the 39th Annual Meeting of the Japanese Society of Spinal Surgery on June 12, 2025.

## Case presentation

Case 1

An 84-year-old woman presented with left leg pain radiating from the anterior thigh to the medial knee for several months. Symptoms persisted despite medication and impaired her ability to walk. She had previously undergone L4/5 and L5/S1 posterior lumbar interbody fusion 10 years earlier.

Physical examination revealed pain radiating along the anterior thigh to the medial knee, with reproduction of symptoms during femoral nerve stretching and Kemp tests. Muscle strength was normal [manual muscle testing grade 5 throughout.

**Figure 1:**
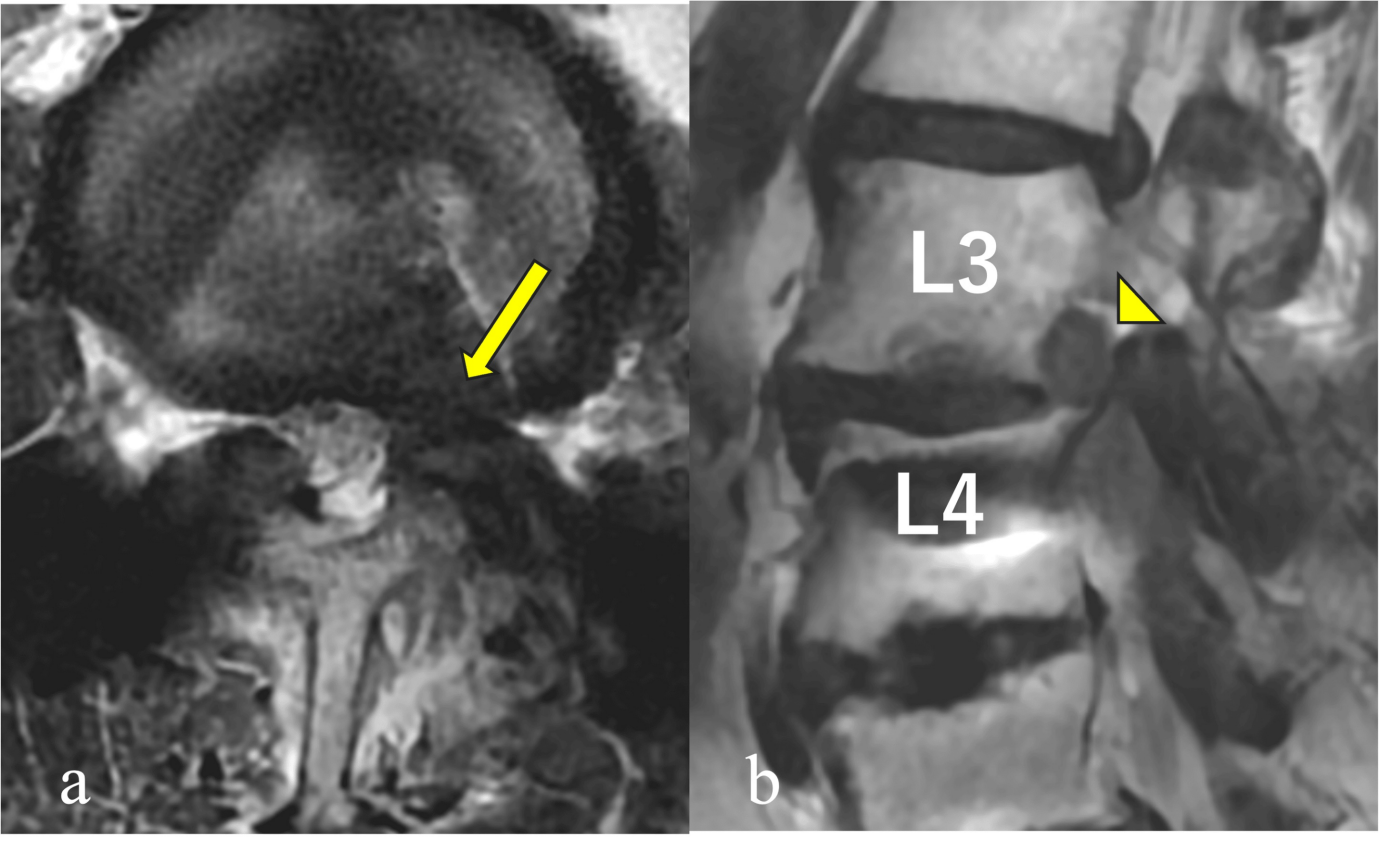
Axial and sagittal MRI findings in case 1 The axial T2-weighted image (a) demonstrates a left L3/4 foraminal disc herniation (arrow), and the sagittal image (b) shows cranial displacement of the L3 nerve root (arrowhead).

MRI revealed a left L3/4 foraminal disc herniation (Figure 1). 

Metallic artifacts from prior fusion at L4/5 and below hindered detailed visualization of nerve branches.

Surgery was performed as described. After confirming the disc bulge, a small amount of dye was injected into the disc to assist in identifying the herniated fragment. While removing fat tissue over the disc using forceps, thin, vine-like, whitish fibers were identified. No changes in MEPs or SEPs were observed. The stained herniated disc was removed, but no neural continuity was visualized proximally, although the distal segment was identifiable (Video 1).

**Video 1:** Intraoperative endoscopic findings in case 1 With the bevel directed laterally, the bulging disc was identified, and the fat tissue overlying the disc was removed using punch forceps. During this process, some vine-like whitish fibers were encountered. No significant changes in motor evoked potentials (MEPs) or somatosensory evoked potentials (SEPs) were observed, and nerve injury was not suggested. The stained herniated disc was then identified and removed. The peripheral side of the vine-like whitish fibers was confirmed.

**Figure 2:**
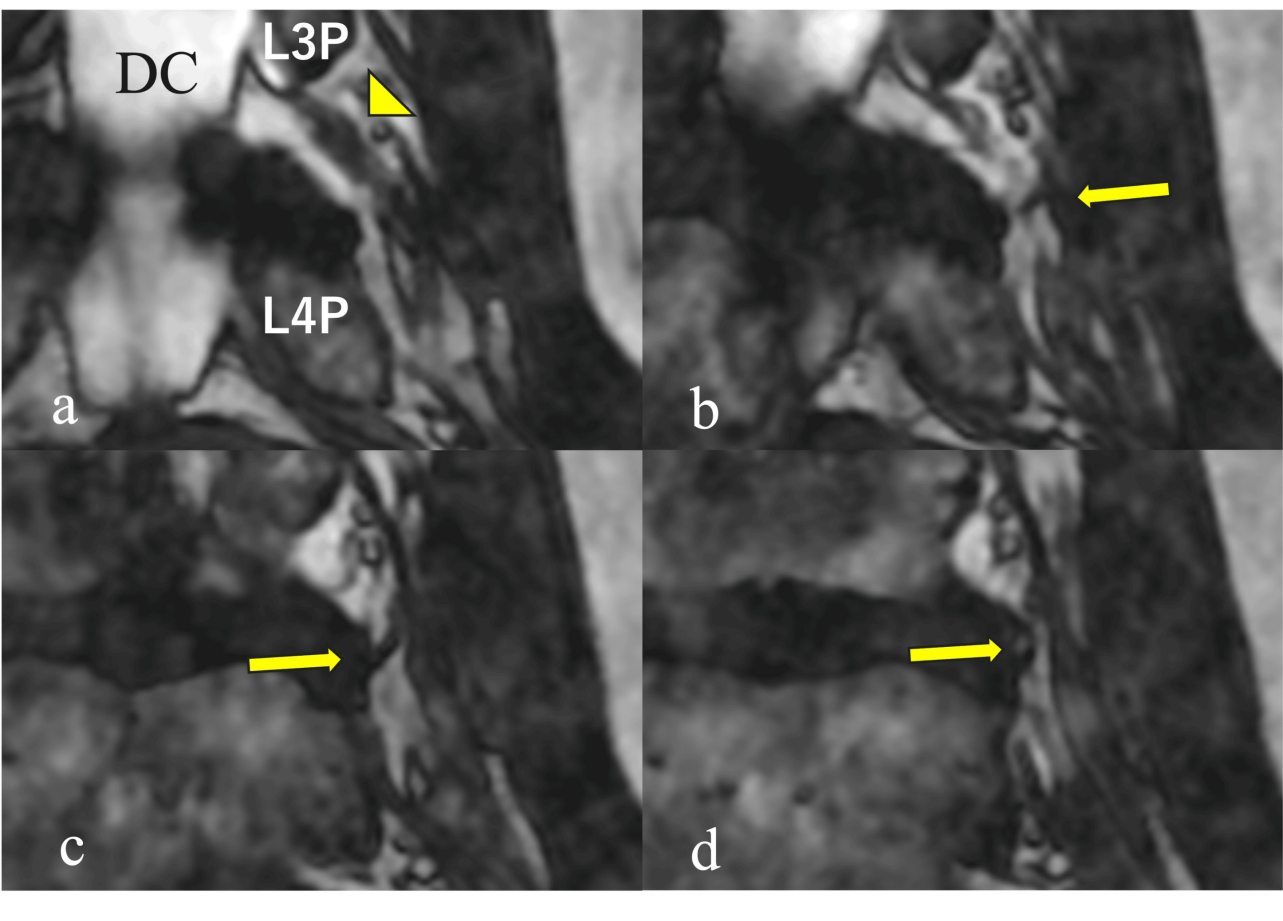
A coronal FIESTA MRI 10 years ago depicting a nerve-like branch arising from the L3 nerve root Coronal FIESTA MRI images obtained before L4–S1 lumbar fusion in case 1 demonstrated a nerve-like structure (arrow) branching from the L3 nerve root (arrowhead) and coursing toward the disc. Panels (a–d) represented sequential coronal slices arranged from dorsal to ventral. (DC: dural canal; L3P: L3 pedicle; L4P: L4 pedicle)

Postoperatively, leg pain and numbness improved immediately. On review of the pre-fusion FIESTA MRI from 10 years earlier, a fine nerve branch arising from the L3 nerve root toward the disc was clearly visible, matching the intraoperative findings (Figure 2).

**Figure 3:**
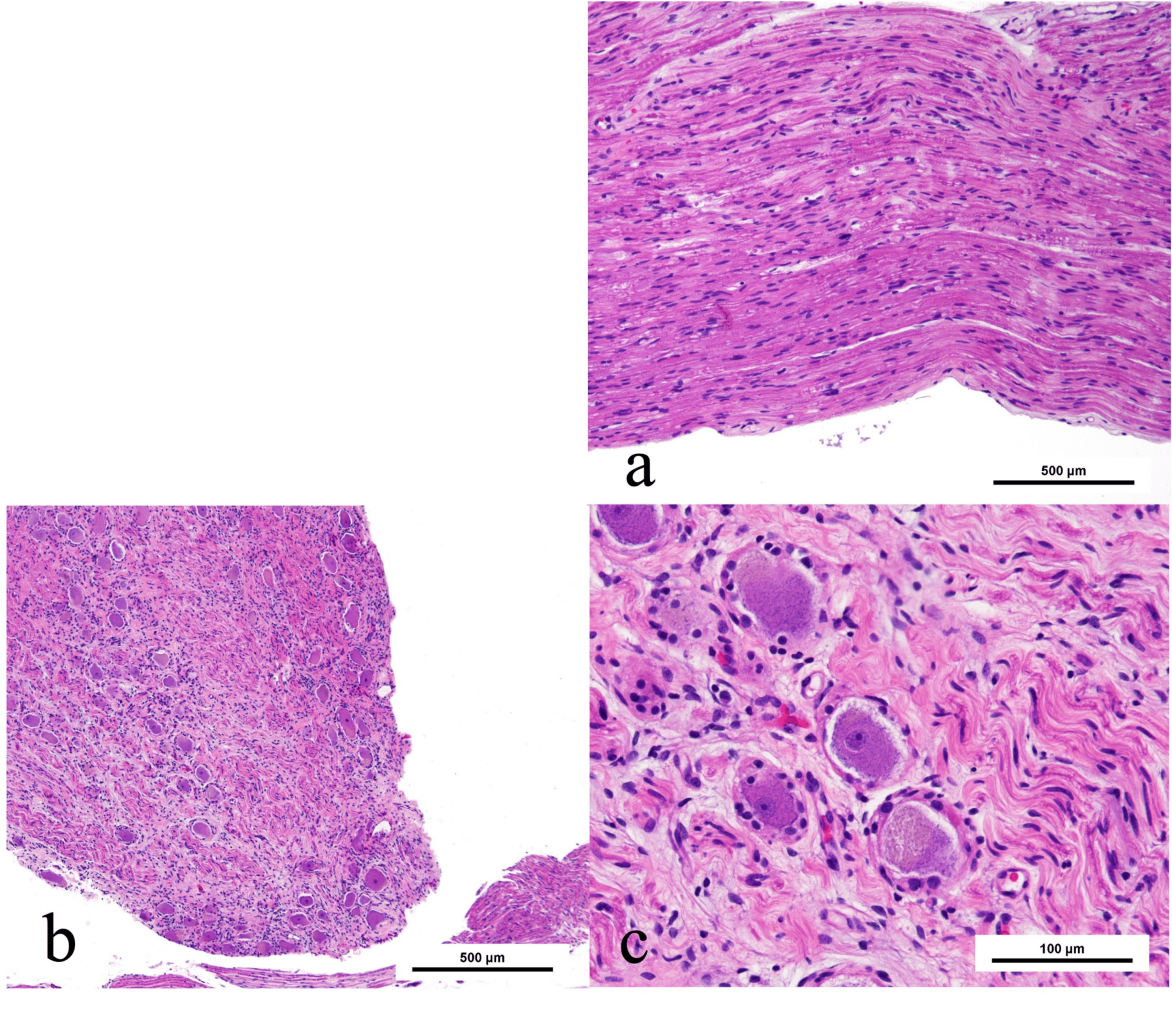
Histopathological features in case 1 Histopathological findings in case 1. Multiple large cells are present in clusters (a), surrounded by small satellite-like cells (b), and wavy, spiral fibrous structures are observed (c), consistent with typical nerve tissue.

Histology revealed clusters of large neural cells surrounded by satellite-like small cells and wavy/spiral fiber architecture, consistent with a peripheral ganglion (Figure 3).

Case 2

A 61-year-old man presented with left anterior thigh pain and low back pain for three months. Symptoms persisted despite conservative treatment. He had difficulty walking due to pain.

Physical examination showed radiating anterior thigh pain with femoral nerve stretching and Kemp tests. Mild iliopsoas weakness (manual muscle testing grade 4−) was observed.

**Figure 4:**
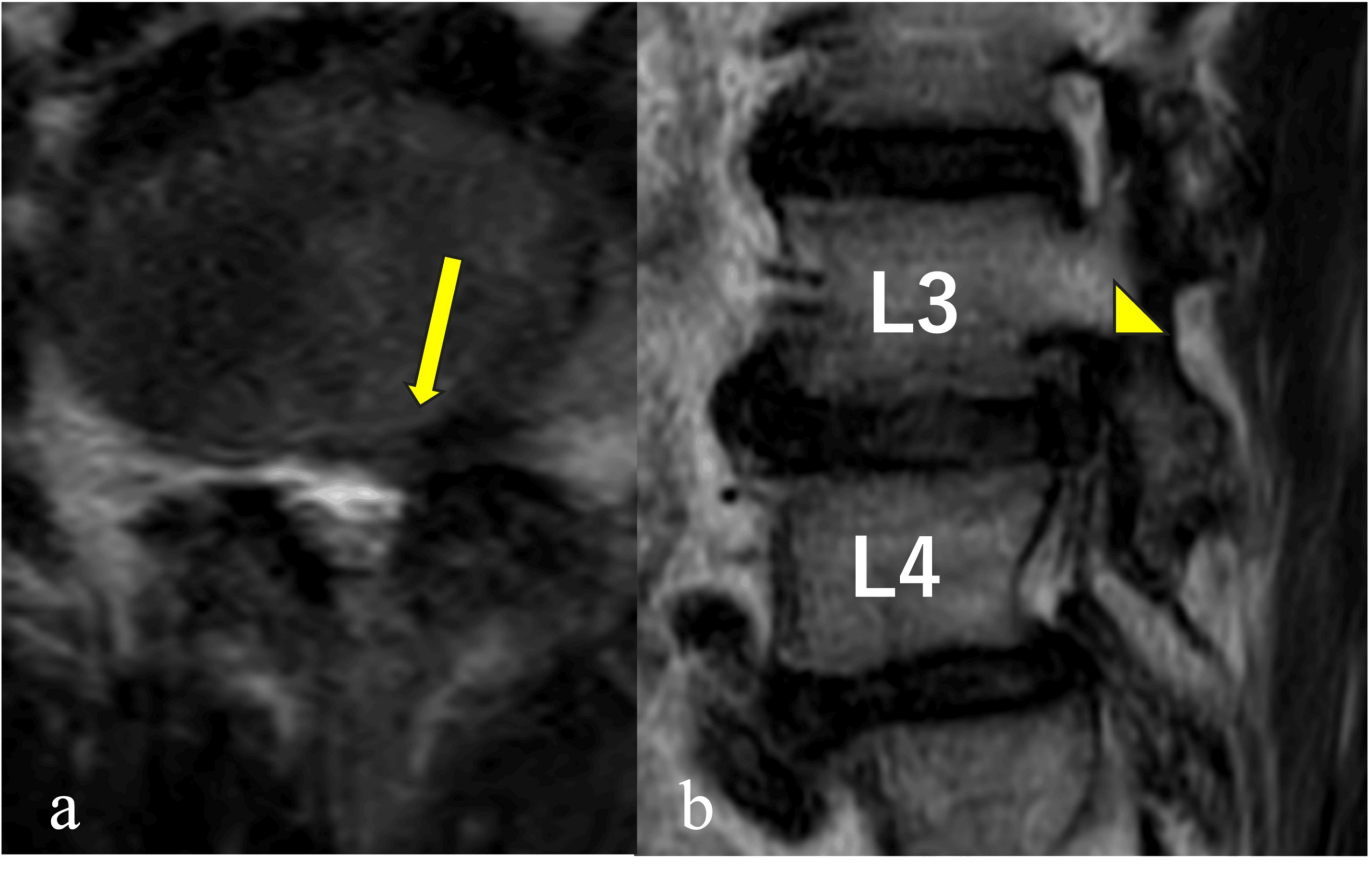
Axial and sagittal MRI findings in case 2 Axial T2-weighted MR image (a) in case 2 shows a foraminal-type lumbar disc herniation at L3/4 (arrow). On the sagittal image (b), the L3 nerve root is displaced cranially (arrowhead).

MRI showed a left L3/4 foraminal disc herniation with cranial displacement of the L3 nerve root (Figure 4).

Surgery was performed using the same outside-in approach. While removing fat tissue over the disc with punch forceps, vine-like whitish fibers were identified. No abnormal MEP/SEP findings were observed. The fibers did not show proximal continuity. The herniation was fully removed, and the nerve root was visualized from proximal to distal without encountering a central continuation of the unidentified fibers (Video 2).

**Video 2:** Intraoperative endoscopic findings in case 2 After confirming the bulging disc with a dissector, the fat tissue overlying the disc was removed using punch forceps, during which vine-like whitish fibers were observed. No abnormal waveforms were detected on MEP or SEP monitoring. Although the fibrous structure was followed proximally, continuity toward the central direction could not be confirmed. The herniated disc located caudal to the nerve root was completely removed, and continuity and pulsation of the nerve root were verified. The nerve root was observed from proximal to distal; however, no proximal continuation of the vine-like whitish fibers was identified.

Postoperatively, symptoms improved markedly, and no new neurological deficits occurred. At one-year follow-up, no recurrence or complications were observed.

**Figure 5:**
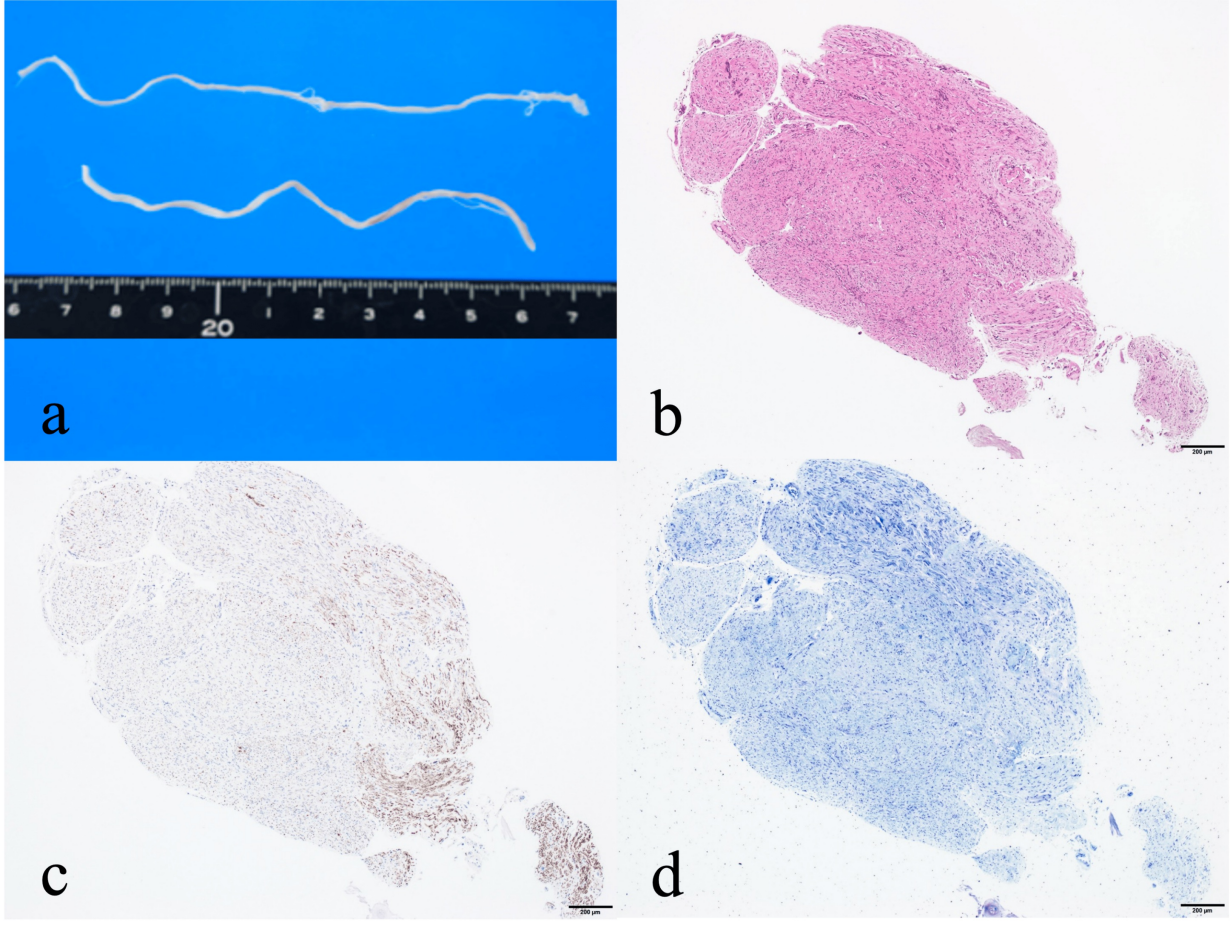
Gross and histopathological characteristics of the resected nerve-like structure in case 2 A gross specimen from case 2 is shown in (a). The specimen appears as a continuous white cord-like structure approximately 10 cm in length. Hematoxylin and eosin (H&E) staining suggests peripheral nerve tissue, similar to case 1 (b). Neurofilament staining confirms the presence of axonal structures (c), whereas Klüver–Barrera staining is negative, indicating the absence of myelinated fibers (d). These findings are compatible with a gray ramus communicans composed predominantly of unmyelinated nerve fibers.

Histology revealed typical neural tissue. Neurofilament staining confirmed axonal structures, while Klüver-Barrera staining was negative for myelin, indicating unmyelinated fibers (Figure 5).

These findings suggested gray rami communicantes.

## Discussion

Anatomical background

**Figure 6:**
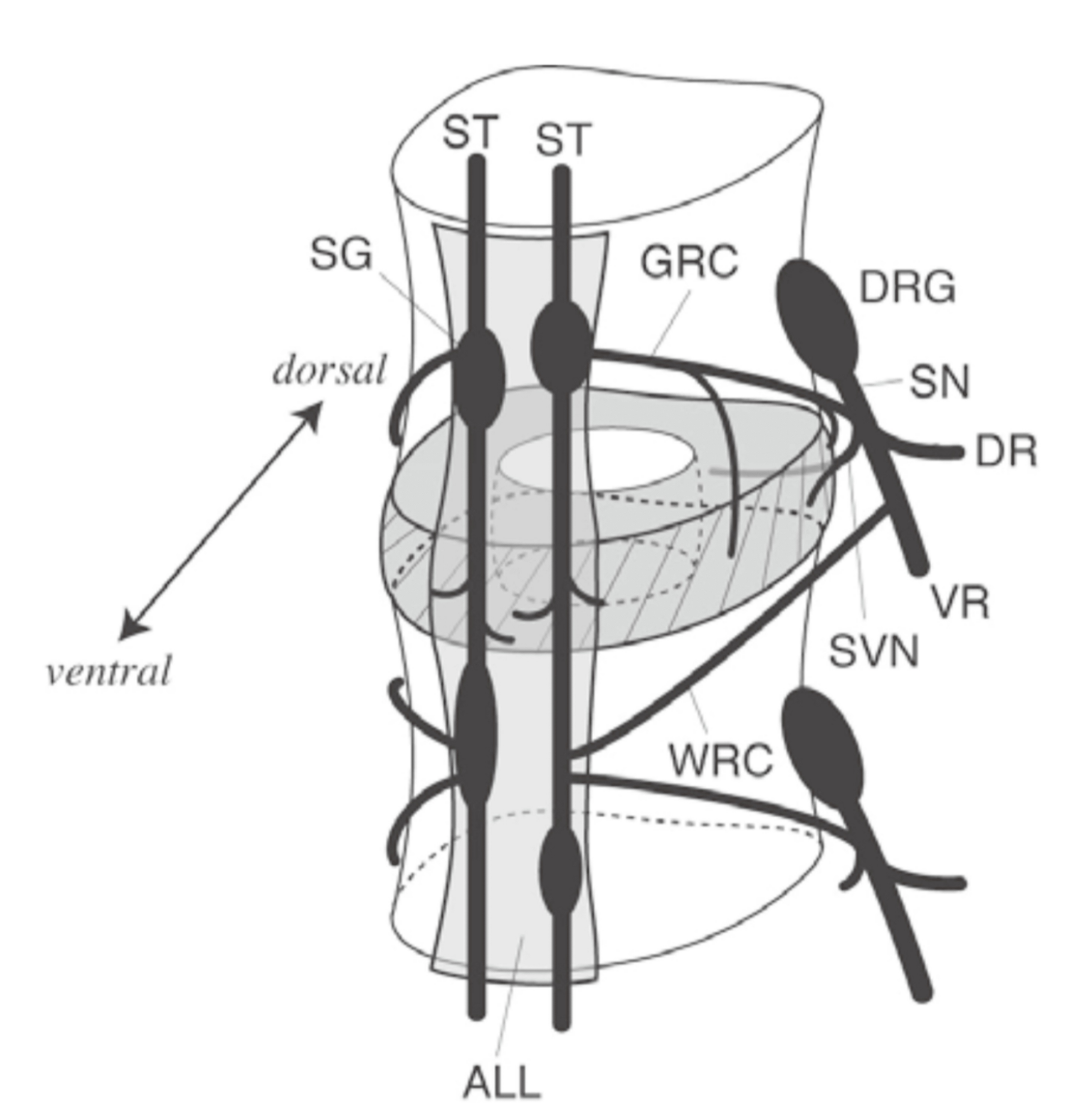
Anatomy of the gray and white rami communicantes Gray and white rami communicantes connect the spinal nerve with the sympathetic trunk. This schema is reproduced from the cadaveric study by Takahashi et al. (DRG: dorsal root ganglion; SN: sinus nerve; DR: dorsal root; VR: ventral root; SVN: sinus vertebral nerve; ST: sympathetic trunk; SG: sympathetic ganglion; GRC: gray rami communicantes; WRC: white rami communicantes; ALL: anterior longitudinal ligament). Adapted from Takahashi et al., 2007 [8], with permission from The Journal of Japanese Society of Lumbar Spine Disorders. Permission obtained.

Gray rami communicantes consist mainly of unmyelinated postganglionic sympathetic fibers running from the sympathetic trunk to the spinal nerves (Figure 6) [8].

They are present at all spinal levels and may be more prominent at the thoracolumbar junction [9]. Their extremely small caliber and blending with surrounding fat and ligamentous tissues make intraoperative identification difficult.

Recent anatomical studies have shown that gray rami communicantes exhibit marked variations in their course near the neural foramen. Some fibers cross the disc level or extend ventrally [10].

Zheng et al. classified lumbar rami communicantes into superficial, deep, and discal branches, including fibers tightly adherent to the disc surface [11], demonstrating that these structures can follow multiple pathways around the foramen and disc.

These variations support the anatomical plausibility of the nerve-like structures observed in our cases. Whether the L3/4 level has a unique predisposition remains unclear and requires further study.

Differentiation from ectopic neural tissue

In the present cases, the nerve-like structures were interpreted as gray rami communicantes rather than ectopic neural tissue based on a combination of intraoperative, anatomical, and histopathological features. Intraoperatively, the fibers were extremely thin, vine-like, and whitish, lacking the typical fascicular architecture of spinal nerve roots. They were located ventral to the nerve root and adjacent to the disc surface, which is consistent with the reported course of discal or deep branches of the gray rami communicantes. Importantly, despite careful proximal and distal exploration, no continuity with the main nerve root or thecal sac could be identified.

Histologically, neurofilament staining confirmed the presence of axons, whereas Klüver-Barrera staining was negative for myelin, indicating unmyelinated fibers. This profile is characteristic of postganglionic sympathetic fibers that constitute gray rami communicantes and is inconsistent with ectopic spinal nerve tissue, which typically contains myelinated fibers and organized fascicles. Taken together, these findings support the interpretation that the resected structures represented gray rami communicantes rather than ectopic neural tissue.

Clinical significance and intraoperative management

When a nerve-like structure appears in an unexpected location, its identity is difficult to determine intraoperatively. Although no neurological deficits occurred after resection in these cases, unnecessary excision should be avoided because of potential functional relevance. Moreover, because gray rami communicantes consist mainly of postganglionic sympathetic fibers, their function cannot be adequately assessed by intraoperative neurophysiological monitoring such as MEP or SSEP, which primarily evaluate somatic motor and sensory pathways. This limitation likely explains why no significant changes in MEP or SSEP were observed after resection in our cases. Gentle handling and, when feasible, consideration of pathological confirmation or postoperative imaging review are recommended when encountering such structures [7].

Preoperative imaging and diagnostic support

In case 1, preoperative coronal FIESTA MRI clearly showed a small branch extending from the nerve root toward the disc (Figure 2). Fine structures such as these can be visualized more readily using balanced steady-state free-precession sequences like FIESTA and CISS. High-resolution 3D MRI using these sequences has been shown to visualize foraminal ligaments and nerve roots in vivo [12-14].

Such findings may serve as important clues indicating the presence of anomalous neural structures, which is especially valuable in endoscopic surgery where the working space is narrow, and the risk of unintentional nerve contact is higher.

Comparison with prior literature

Reports of gray rami communicantes directly visualized during endoscopic lumbar surgery and histologically confirmed are exceedingly rare. Although Ruetten et al. noted nerve-like structures during transforaminal endoscopic procedures [7], histological confirmation was generally lacking. Thus, the present cases provide valuable evidence supporting the existence and surgical relevance of these structures.

Limitations and future perspectives

This report is based on two cases. Further clinical reports, cadaveric studies, and high-resolution imaging using FIESTA or CISS are necessary to clarify the prevalence, variations, and three-dimensional anatomy of these fine neural structures. Incorporating recognition of such nerve variations into preoperative imaging algorithms may enhance surgical safety.

## Conclusions

We report two cases in which unexpected nerve-like structures not corresponding to typical spinal nerve root anatomy were encountered during endoscopic lumbar discectomy and were histologically confirmed as neural tissue. These findings highlight the possibility of diverse small neural structures, including gray rami communicantes, around the intervertebral disc, which may enter the operative field during endoscopic procedures.

Understanding inter-individual anatomical variations and avoiding misidentification of ectopic or branching neural fibers are essential to minimize neural injury. Accumulation of intraoperative observations combined with histopathological verification, as in the present cases, will deepen understanding of the neural structure surrounding the intervertebral disc and may contribute to refining surgical safe zones and imaging strategies.

## References

[1] Yokosuka J, Oshima Y, Kaneko T (2016). Advantages and disadvantages of posterolateral approach for percutaneous endoscopic lumbar discectomy. J Spine Surg.

[2] Kambin P, Sampson S (1986). Posterolateral percutaneous suction-excision of herniated lumbar intervertebral discs. Report of interim results. Clin Orthop Relat Res.

[3] Kambin P, Brager MD (1987). Percutaneous posterolateral discectomy. Anatomy and mechanism. Clin Orthop Relat Res.

[4] Lewandrowski KU (2020). The strategies behind "inside-out" and "outside-in" endoscopy of the lumbar spine: treating the pain generator. J Spine Surg.

[5] Yeung A, Lewandrowski KU (2020). Five-year clinical outcomes with endoscopic transforaminal foraminoplasty for symptomatic degenerative conditions of the lumbar spine: a comparative study of inside-out versus outside-in techniques. J Spine Surg.

[6] Choi G, Pophale CS, Patel B, Uniyal P (2017). Endoscopic spine surgery. J Korean Neurosurg Soc.

[7] Ruetten S, Komp M, Merk H, Godolias G (2008). Full-endoscopic interlaminar and transforaminal lumbar discectomy versus conventional microsurgical technique: a prospective, randomized, controlled study. Spine (Phila Pa 1976).

[8] Takahashi Y, Ohtori S, Aoki Y (2007). Basic studies relevant to discogenic low back pain. Jr Jap Soc Lum Spi Dis.

[9] Standring S (2020). Gray’s Anatomy: The Anatomical Basis of Clinical Practice.

[10] Zhao Q, Cheng L, Yan H (2020). The anatomical study and clinical significance of the sinuvertebral nerves at the lumbar levels. Spine.

[11] Zheng Z, Ma R, Zhang R (2022). Anatomical study and clinical significance of rami communicantes of the lumbar spine. Reg Anesth Pain Med.

[12] Li Z, Chen YA, Chow D (2019). Practical applications of CISS MRI in spine imaging. Eur J Radiol Open.

[13] Henkelmann J, Wiersbicki D, Steinke H (2022). In vivo detection of the lumbar intraforaminal ligaments by MRI. Eur Spine J.

[14] Ramli N, Cooper A, Jaspan T (2001). High resolution CISS imaging of the spine. Br J Radiol.

